# Tensions regarding inclusion meanings from stakeholders’ perspectives: A qualitative study

**DOI:** 10.1371/journal.pone.0321375

**Published:** 2025-04-07

**Authors:** María-José Solis-Grant, María-José Bretti-López, Cristóbal Sepúlveda-Carrasco, Cristhian Pérez-Villalobos, Tatiana Sepúlveda-Witt, Eduardo Reinoso-González, Juan Francisco Hernández-Quidel

**Affiliations:** 1 Departamento de Kinesiología, Facultad de Medicina, Universidad de Concepción, Concepción, Biobío, Chile; 2 Universidad de Concepción, Concepción, Biobío, Chile; 3 Exercise and Rehabilitation Sciences Institute, School of Occupational Therapy, Faculty of Rehabilitation Sciences, Universidad Andres Bello, Santiago, Chile; 4 Medical Education Department, School of Medicine, Universidad de Concepción, Concepción, Biobío, Chile; 5 Universidad de Los Andes, Santiago, Las Condes, Chile; Università Ca' Foscari, ITALY

## Abstract

In literature, a divergence is apparent concerning the definition of educational inclusion. The study aims to describe the relationship between the diversity of actors in the university community and their perception of institutional inclusion in higher education organizations in Chile. We employed a qualitative methodology based on Grounded Theory, conducting focus groups with 16 students, 17 teachers, five non-teaching staff, and eight authorities. We conducted a semi-structured interview with an authority, encompassing all three university campuses in the country. The data analysis revealed five subcategories associated with the topic. What is understood by the concept of inclusion? What do they think about inclusion? Who should be the subjects of inclusion? What changes have been observed in inclusion? Moreover, why is it necessary to define inclusion? The emphasis is on the diverse existing views around the approach to the concept and the need to generate definitions with institutional relevance to advance its development and implementation.

## Introduction

Educational inclusion is an ongoing process linked to social justice and equity, involving enriching educational centers through the dynamic and achievable development of inclusive values, policies, and practices [1]. However, there needs to be more consensus regarding to this concept, with various conceptualizations coexisting concerning what inclusion is and what characteristics define inclusive actions in the educational sphere [2]. This conceptualization is due, on one hand, to the fact that the object of exclusion or source of discrimination varies over time and space [3] and, on the other hand, due to the progressive recognition of groups that have historically been excluded or marginalized and the blending of internal or socially perceived identities that a person may have, such as gender, age, disability, race, ethnicity, religion, and sexual orientation [4]. In this way, it is possible to find various approaches and conceptualizations, depending on the characteristic of the human being that is the focus of discrimination. For example, in Latin America, the initial advancements in the concept of educational inclusion began with an “integration” approach, dating back to the late 19th and early 20th centuries with the emergence of school education systems seeking to integrate native and immigrant residents. However, this theoretical approach was challenging to implement, and exclusionary practices began to emerge, primarily due to school dropout and repetition, occurring mainly in students from socioeconomically vulnerable sectors, posing a difficulty that needed to be overcome; to address that, a series of strategies were implemented [5]. First, there was a period of assistance in the 1940s and 1950s, providing students with essential elements and materials to ensure their access to education, such as food, clothing, and school supplies. Second, in the 1960s and 1970s, strategies focused on learning difficulties and the characteristics of students facing such difficulties. Thus, “psychopedagogy” emerged in response to the development of education sciences, particularly psychology, and psychopedagogy. From this perspective, responsibility for failure is once again attributed to the student, leading to the creation of special education centers. Thirdly, compensatory policies emerged between the eighties and nineties, stipulating a shift in perspective by focusing on the educational center, considered the core of the unit of change and problem resolution. Finally, the inclusion strategy emerges today, indicating two different positions: one that understands inclusion as equity or equal opportunities for access, retention, and graduation from the school system, and another that considers inclusion to be equity understood as providing each student with what is necessary to meet their particular needs [5].

In general terms, globally historically, concepts related to educational inclusion have been linked to individuals with disabilities, initially using the concept of normalization, which is considered the guiding principle of the integration concept. Bank-Mikkelsen, the director of the “Danish Service for Mental Retardation,” forged this term in 1959 [6]. His statements referred to the idea that adults with intellectual disabilities should lead an everyday life like others, thus reinforcing their fundamental human rights [6]. Subsequently, the term spread across Europe, conceptualizing the principle of normalization as the right of individuals with intellectual disabilities to have living conditions similar to those of the broader society [7]. In the following decades, new proposals and approaches emerged regarding this concept: ways of understanding the world that, above all, require the recognition of differences and the inclusion of each individual in the society, considering all dimensions of the human being, including but not limited to the condition of disability [8–10].

The conceptualizations presented so far have origins in the lower levels of education; nevertheless, some of these concepts and their strategies have been adapted and implemented in higher education to promote student access and retention [11,12].

In regard of the historical review of the concept, authors Ainscow, Booth, and Dyson [13] assert that one can differentiate definitions into two broad groups: narrower ones that focus on students with disabilities, the placement of certain students, and the social and academic diversity needs of specific student groups; and broader and more recent ones that shift their focus to identifying how educational institutions can provide support to all their students [14]. Moreover, Tiernan [15] argues that distinguishing between inclusion and total inclusion is essential for developing a clear vision of inclusive education. Within this distinction, inclusion can be understood as meeting the needs of students with special educational needs in mainstream education, when possible, with separate environments considered as an option when appropriate, on a continuum of services [16]. Thus, special schools and special classes are synonymous with providing more significant support for students educated in these environments. While this interpretation aims to ensure the right to education for all, it also creates dilemmas of inequality within the system. It provides access to education but also promotes positive discrimination, as it is subject to debates and different perspectives based on traditional education models. This is because it relies on the identification and assessment of individual needs. On the other hand, we can understand total inclusion as including all students in mainstream education [17]. In this context, the existence of segregated environments is deemed exclusionary, and education outside conventional classrooms is not considered legitimate. Advocates for total inclusion argue that this conception destabilizes ableism and places diversity at the core of an educational system, thus promoting the development of inclusive societies. However, not everyone universally accepts total inclusion and questions it as the only model for delivering services to students with complex needs in regular schools. Advocates argue that while ensuring access and participation in education alongside peers is crucial, these should not be at the expense of eliminating opportunities for specialists to provide intense, individualized, and explicit instruction and interventions [18]. This debate reflects the division between philosophical rhetoric and the practice of inclusive education [15]. Goransson and Nilhom [19], in their analysis of documents between 2004 and 2012, identify four qualitatively different categories of school inclusion definitions. Category A includes students with disabilities in regular education classrooms. In Category B educators meet the social and academic needs of students with disabilities individually. Category C focuses on meeting the social and academic needs of all students, while Category D involves the pursuit of creating inclusive communities [14]. According to the framework proposed by Ainscow, Booth, and Dyson [13], Categories A and B correspond to narrow definitions, and Categories C and D align with broader ones [14].

On the other hand, Ainscow and Miles [20] acknowledge other sources of heterogeneity in the conceptions of school inclusion found in the literature. First, inclusion concerning disability and the special educational needs of students. Second, understood as a response to the partial or total withdrawal of a student from their educational context, also known as disciplinary exclusion. Third, focused on the inclusion of vulnerable groups. Fourth, inclusion understood as a driver for a school for everyone, and fifth, inclusion is Education for everybody [21]. The latter corresponds to a formalized proposal in the Salamanca Statement (1994) that promotes integrating and appreciating students with their unique characteristics and needs, ensuring effective learning [22]. Its impact lies in ensuring access to education for socially excluded, marginalized, and discriminated groups, laying the foundation for inclusive education at all levels [23].

In order to achieve education for all, international organizations have developed guiding documents for inclusion, such as the “Guidelines on Inclusion Policies in Education” by the United Nations Educational, Scientific and Cultural Organization from 2009 and the “Guide to Inclusive Education: Developing Learning and Participation in School Centers” by the Organization of Ibero-American States for Education, Science, and Culture in collaboration with the FUHEM Foundation [24]. In Chile, educational inclusion operates within the legal framework of various laws, representing progress in the Chilean educational system. Some laws are specific to the school level, such as Law No. 20.845, known as the Inclusive School Law [25]. Others are cross-cutting and apply to any area of participation, including all educational levels. Examples include Law 20.422 on equal opportunities and social inclusion for people with disabilities [26], Law 20.609, establishing measures against arbitrary discrimination [27], and Law 21.545, promoting the inclusion, comprehensive care, and protection of the rights of individuals with autism spectrum disorder in the social, health, and education spheres [28]. Finally, there is also a specific law for higher education level, corresponding to Law 21.091 on higher education. Among its articles, the law establishes the promotion of inclusion and the elimination of discrimination, the right of individuals to pursue this educational level, the effective participation of all individuals in the institution, and the implementation of reasonable accommodations when needed [29].

An unanimously agreed-upon aspect in literature is that the concept of inclusion is related to the concept of diversity, which refers to the combination of characteristics, experiences, and competencies that make individuals unique, adding value to communities [30]. In other words, it involves individual, social, and cultural differences and implies the right to express and respect them [31]. Similarly, inclusion promotes awareness and acceptance of diversity and the appreciation of individuality [32]. Consequently, inclusion refers to respecting all individuals’ human rights facilitating their full participation in community life [33]. Therefore, inclusive education may be defined as a philosophy and educational practice that aims to ensure the learning of students and their full participation. It is also a process and system of beliefs that seeks to challenge exclusion in educational systems [34]. Currently, there are proposals regarding the definition of inclusion in higher education, such as that of Brito, Basualto, and Reyes [35], who present inclusion in higher education from a social and educational perspective. They propose a typological definition encompassing belonging, equality, equity, and social justice—dimensions that should be present in educational policies; otherwise, inclusion will paradoxically remain exclusionary (p.157).

Currently, one of the main challenges that higher education institutions face when declaring themselves inclusive is to materialize their conception and approach in their institutional documents [36]. It is also noted that educational inclusion faces evident tensions between rhetoric and practice [15].

One can understand the above because the concept of inclusion implicitly includes exclusion, creating a paradox where equity and integration may be affected, potentially leading to segregated practices [37].

Considering these conceptual tensions about inclusion in education in general and in higher education specifically, it is necessary to understand how university community members conceptualize this concept to provide empirical evidence from the contexts in which they expect to implement it. For this reason, this research aims to describe the relationship among diverse actors within the university community regarding their perception of institutional inclusion in higher education organizations in Chile.

## Materials and methods

The Ethics, Bioethics, and Biosafety Committee of the Universidad de Concepción in Chile approved the present research (CEBB 703–2020). The recruitment period, started on December 23rd 2020, and ended on March 17th 2021.

We conducted the study at a university in Chile, located outside the country’s capital. This university receives direct state funding and manages the standardized process of student selection at the national level in Chile.

The study employed a qualitative methodology, which allowed to address phenomena from the perspective of the individuals involved [38] and was based on Strauss and Corbin’s grounded theory [39,40] to connect with the multiplicity of perspectives of the actors around the phenomenon under study; developing theories and concepts based on the systematic collection and analysis of data. The data production techniques included the Focus Group and the Semi-Structured Interview. After obtaining individual informed consent, we conducted and recorded the sessions using the online meeting platform Zoom©.

Each participant received a written informed consent form before the focus group. They had to print and sign the form and then submit it to the researchers. The researchers verbally repeated the informed consent key topics at the beginning of the focus groups. They explained to the participants: the purpose of the study, the kind of participation requested, the guarantee for free and voluntary participation, the confidentiality of their data, and their right to withdraw without consequences.

Two trained in qualitative methodologies psychologists conducted the interviews, using thematic scripts generated by the research team, and developed field notes during each session, which lasted between 60 and 90 minutes.

The participants were members of the educational community who belong to the four categories (students, authorities, teachers, and non-teaching staff) and the three campuses of the University. As an inclusion criterion among the students, those who were regular students in the semester of the focus group application were considered. For authorities, individuals with responsibilities for a unit of institutional management recognized by the organizational chart and who had staff under their supervision were considered. In relation to the teachers, inclusion criteria covered professionals contracted to teach in undergraduate and postgraduate programs of the institution. For non-teaching staff, both professional and non-professional personnel hired for roles other than teaching were included.

We used a non-probabilistic convenience sampling method through an open call by category. Subsequently, upon agreement by the research team, we utilized theoretical sampling, selecting cases based on their potential contribution to the ongoing development and refinement of the evolving theory. Participants were included until achieving theoretical saturation, as indicated by the inclusion of 40 participants. We disaggregated the categories into five groups: undergraduate students, postgraduate students, authorities, teachers, and non-teaching staff. The researchers conducted separate meetings with each group.

14 undergraduate students, 2 postgraduate students, 15 teachers, 5 non-teaching staff, and 8 authorities participated in the focus groups. Additionally, 1 authority took part in the semi-structured interview. Concerning gender distribution in the total sample (N = 47), 24 (51.06%) were women, 22 (46.81%) were men, and 1 (2.13%) identified as non-binary. Regarding diversity, 2 individuals identified as neurodivergent [41], 2 students had visual disabilities, and 1 person identified as part of the sexual dissidence.

Concerning the knowledge disciplines [42] represented in the sample, participants included those from Natural Sciences, Engineering and Technology, Medical and Health Sciences, Social Sciences, and Humanities. Each participant received a single invitation to each meeting.

The data analysis involved conducting an open coding process, enabling the identification and description of categories inductively. The researchers utilized the data analysis software ATLAS.ti [43].

To ensure the reliability of the study, we implemented strategies to guarantee an interpretive agreement of the data. These strategies included the triangulation of researchers through regular meetings among the coders, with the aim of discussing and contrasting their codifications, complementing and deepening the data analysis. Additionally, records of agreements were maintained with the goal of having this input to review the information throughout the coding and analysis process. Similarly, meetings with the entire research team were conducted to share findings and progress, which allowed for a deeper analysis, achieving a better understanding of the collected data.

## Results

From the totality of results obtained in the qualitative phase of the study, this section presents five subcategories associated with the conceptualization of inclusion. These results emerged from the reading and analysis of the transcribed documents. We openly presented them to the general community through an online conference on the Zoom© platform, which included a space for comments; we considered no further modifications necessary despite the opinions received.

In the following section, we will describe the subcategories, illustrating them with direct quotes extracted from the transcripts and accompanied by the abbreviation “F. G” for focus group, “I” for interviews, and “P” for participants, along with an Arabic number indicating the participant number, followed by their role and gender.

## Inclusion conceptualization

### 1. What is understood by the concept of inclusion?

Participants posit inclusion as the human right of every person to be part of a group, manifested in practices associated with respecting and valuing diversity. Concerning this last one, in the educational realm, participants emphasize the need to recognize and appreciate differences among individuals, given the potential this offers to enrich common teaching and learning spaces.

“...recognizing the differences of each individual and precisely valuing that these differences do not make us less competent or less suitable for something (...) these differences give us a different perspective, a different worldview, which allows us to enrich, in general, what the common processes are.” [F.G., P1, undergraduate students, male]

On the other hand, interviewers note that inclusion encompasses everything that prevents processes of exclusion, which, from one perspective, humans inevitably create due to the need to construct differences. In this way, inclusion would constitute regulations or guidelines that society arbitrarily establishes to avoid such exclusionary processes.

“If I want to generate a norm for inclusion, a sociability that builds inclusion based on processes because I speak disciplinarily, the processes of exclusion are inevitable, they are inevitable, you need to construct a difference, you need to have people who fall outside your parameters. How far will you take that to the extreme of guiding your daily relationships with people? That’s what needs to be resolved in terms of society.” [F.G., P3, authorities, male]

Similarly, individuals refer to inclusion as eliminating barriers that inhibit a person’s full development through institutional and individual adaptations, considering their specific abilities.

“Because many people think that inclusion is about making life easy for the other person, and it’s not that, but rather adapting and also, that the institution adapts to the disability or any kind of problem the student may have (...) that both can adapt.” [F.G., P1, undergraduate students, male]

Finally, in the participants’ opinion, the definition of inclusion is related to concepts such as justice, equity, and equality in the conditions for the development of each person and the possibility of participation for everyone, regardless of the nature of their abilities.

“I understand it as the acknowledgment, or rather, the recognition that not all of us are standing on the same level, but for example, some need more help to be on an equal footing, so to speak, to be on an equal basis with perhaps a group or an individual in a certain hegemony.” [F.G., P2, undergraduate students, non-binary].

### 2. What do they think about inclusion?

Some participants mention feeling ignorant, lacking perspective, and having doubts or clarity about it. Additionally, participants note that it is a concept that needs to be integrated and new for some people, highly susceptible to changes over time, relatively broad, and ambiguous.

“Personally, I don’t have the concept very well integrated. I believe it’s a concept that may be relatively new for many.” [F.G., P1, teachers, female]

“I don’t know if there would be a better one, and I don’t dare to criticize it either. But what I do believe, and I think we all agree on this, is that it’s super ambiguous; it doesn’t say anything; I stay in the same place. If they talk to me about inclusion, I stay in the same place. To whom, in what, towards where, what are its limits, its boundaries?” [F.G., P3, authorities, male]

Additionally, there is a perception that inclusion is still seen from a paternalistic and benevolent perspective, contrary to a human rights paradigm; this can lend itself to situations of positive discrimination towards some minority groups. Because of that, certain opinions suggest replacing the concept of inclusion with “good treatment” within a “culture of good treatment” associated with respect for everyone. In this regard, a participant emphasizes that good treatment would mean “the same treatment” for everyone.

“I feel that the concept should be like good treatment. Maybe with other words, but I believe there has to be a culture of respect for everyone (...) I had a case once of a student who was applying to the university, and she had a disability, but she didn’t have economic problems, nothing, she only had something physical (...) And she asked me, ‘How much does the university pay me for having a disability?’ So, what I mean is that it goes beyond that, it’s a culture that comes from the outside.” [F.G., P1, authorities, female]

In this way, the participants perceive a very reductionist view of the concept, stating that it encompasses a broader and deeper scope than what people generally identify as inclusion. They comment that most people think of it only from the perspective of disabilities, often not considering other areas such as gender, sexual, territorial, ethnic, and socio-economic diversities. Because of this, there is a proposed need to surpass the temptation of the bias that limits inclusion only to people with disabilities and to expand the vision of what we will understand as inclusion.

“I would open up the vision of inclusion a little more because I sense that inclusion issues may have been very restricted or rather associated with talking about people with disabilities. I get the impression that maybe they have been very trapped in highlighting those issues, which I think is good, but I would expand the notion of inclusion to other groups that have traditionally and socially been excluded from social participation. I refer, for example, to indigenous peoples, I refer to sexual dissidents, I refer to people with mental health problems, I refer to migrants...” [F.G., P4, teachers, male]

### 3. Who should be the subjects of inclusion?

Concerning this matter, participants hold different opinions. On the one hand, they emphasize that discussing inclusion should not involve making distinctions or segregating based on characteristics. Several interviewed teachers expressed surprise at the question of who should be subjects of inclusion, given the evident differences in the abilities of all individuals. They assert, therefore, that discussing inclusion should involve considering all people.

“Regardless of this classification of categories, of people who require assistance or who have different abilities, we all have different abilities. Probably if we look at each other, we don’t have the same abilities.” [F.G., P6, teachers, female]

On the other hand, some opinions propose directing inclusion towards historically marginalized groups. Correspondingly, the participants assert that inclusion should encompass all who do not feel part of the majority or have different physical or psychological abilities. Additionally, the participants highlight specific considerations regarding inclusion, emphasizing the importance of creating safe spaces primarily for oppressed individuals while respecting and tolerating limits.

“Inclusion precisely aims at those groups that have been marginalized because, from that ‘all’ from that totality, there is a large number of people who are always part of it, who always have the instances, spaces, infrastructure, and everything suitable for their condition. Therefore, when we consider who should be included, precisely, they should be those groups that, for various situations and reasons, are marginalized or have their access to spaces, in this case, the university, not entirely guaranteed.” [F.G., P1, undergraduate students, male]

“Like the paradox of intolerance that is out there, that if we tolerate intolerance, then tolerance itself ends (...) As if I harm others, then maybe I should reconsider that a bit.” [F.G., P2, undergraduate students, non-binary]

Also, other opinions mention not having clarity regarding who should be subjects of inclusion due to the need to realistically consider how much the institution can encompass in terms of concrete actions to address difficulties in this matter.

“And here, I’m not clear about it. Sometimes, I think about groups that are mostly excluded because the issue is the following: people who have difficulties accessing inclusion—meaning the groups are endless; that’s the truth. So, is the university being realistic? How far can it take care of everyone?” [F.G., P3, teachers, female]

Finally, when discussing who should be the subjects of inclusion, participants mention and question the concept of normality as a category that establishes a pre-established norm regarding people’s expected or acceptable difficulties. This leads to the disregard and neglect of various life challenges, such as those related to people’s mental health. The concept of normality also implies its opposite, abnormality, with a generally pejorative connotation. According to the participants, the individuals intended for inclusion would not be abnormal but rather possess different abilities or circumstances.

“I associate the term ‘abnormal’ with a vocabulary that was used at some point with mental retardation, disabled, or terms like valid and invalid, a person who is not worth anything, a person who is retarded. So, that language seems inappropriate to me. Basically, I don’t use the term ‘normal’ because it opposes the abnormal.” [F.G., P3, teachers, male]

### 4. What changes have been observed in inclusion?

Participants mention that, in recent years, this and other topics that were taboo or not assumed as necessary for society have become evident. They argue that the widespread raising of voices in recent years has drawn more attention to and visualized certain situations that affect people not part of the majority, making inclusion a central theme for building a modern society and university today.

“The fact that these groups, let’s call them traditionally marginalized, today have set aside this sense of resignation, which I don’t believe was actually resignation but rather invisibility by the rest of us who were not marginalized, and they begin to make themselves visible and express themselves.” [F.G., P5, undergraduate students, male]

“So, I believe it’s more because of that because people with disabilities are like saying, *“Hey, here we are, we can do the same as my classmate, just in a different way, that’s it, I’m not a weirdo.”* And it also goes hand in hand with the various discriminations we’ve suffered over the years, I mean, there comes a point where people get angry and obviously want to say, *“Let’s cut this out because we are not freaks.”* [F.G., P1, undergraduate students, male]

Additionally, in recent years, there has been an impact on national policies regarding disability due to a change in mentality and thinking about specific issues at the social level. This change is combined with the opinion that younger generations are actively changing paradigms, valuing, and respecting differences much more than previous generations.

“In recent times, especially students, have been promoting inclusion, advocating for the inclusion of everyone, whoever they may be. They want equal opportunities for all people, regardless of their condition (...) I believe that today they are strongly influencing the push for new national policies, in the change and vision we have as a country, and in the commitment, we must have to inclusion.” [G.F., P2, authorities, female]

### 5. Why is it necessary to define inclusion?

Participants agree that, to advance in the institutional implementation of inclusion, the university must first define what inclusion means at its level and agree on the perspective from which to address it. This process aims to establish a common axis throughout the university community.

“I believe that the first step is that we must first establish a common speech as a university. For us, inclusion will be approached from a specific perspective, right? I mean, setting ourselves as a baseline, regardless of the faculty, where certain concepts and definitions come into play.” [G.F., P5, teachers, female]

“I believe that the issue of definitions is super important, and specifying them, I think it is relevant to know institutionally what we are going to understand as an institution, or from what perspective we are going to see it, whether from the assistance, the experiential, or the interaction among the various actors of the university.” [G.F., P2, authorities, male]

They also believe that this concept of inclusion should result from a collaborative, dialogued, and consensus-driven effort by the entire educational community of the University. This would clarify the limits and areas of action regarding the approach to the inclusion theme at the institutional level, which must be a conscious and feasible decision.

“An inclusive university would have to make sure that all its members are inclusive, understand what it means, and come to an agreement or accept inclusion norms (…). That’s why we have to undertake the construction, an arbitrariness that needs to be built in political terms (…), not because we all agree 100%, but because we reach certain consensuses to avoid certain forms of exclusion.” [G.F., P3, authorities, male]

“There has to be a clear definition, a clear guideline that essentially positions the institution at a baseline or starting point, to say: ‘Look, this is the direction we want to go, right? What we aim to achieve, we declare ourselves inclusive because this is what we want to do. We are willing for this, and this is what we can contribute.’” [E1, authorities, female].

## Discussion

Based on the analysis of results, it is possible to recognize that the conceptualizations of inclusion shared by those who participated in the study follow a broad approach, using the terminology proposed by Ainscow, Booth, and Dyson [13]. In other words, they focus on how the institution should incorporate support and consider all students’ social and academic needs [14]. However, in many of the participants’ accounts, it is observed that this approach goes beyond the student body, and it is necessary to consider all individuals in the educational environment. This aligns with the recommendations of various authors suggesting the involvement of all educational community members in inclusive processes [44–46].

Similarly, educational community members reach a consensus on the necessity to expand the concept of inclusion. They perceive a tendency to reduce it solely to the perspective of disabilities, overlooking other types of existing diversities. This consensus proposes a change towards a more holistic view in the hierarchy of inclusion definition categories presented by Göransson and Nilhom [19]. This progression involves moving from a definition of inclusion that understands meeting the social and academic needs of students with disabilities to one that addresses all students’ social and academic needs. This aligns with Tiernan’s [15] “total inclusion” proposal. One can understand it as including all students, including those with complex needs, in general education [17].

However, it is essential to highlight that despite the need to expand the focus of the inclusion concept and the provision of definitions that encompass all members of the educational community, valuing the existing diversity within it, there is disagreement regarding opinions on who should be the subjects of inclusion. In this regard, it is argued on one hand that inclusion should target historically marginalized groups, all those who do not feel part of what is called the majority, and/or those with different physical or psychological abilities. This aligns with Grossman’s [47] perspective on inclusion as a way societies address difference as part of a broader discourse on policies of difference or identity politics, in which politically marginalized groups fight to earn a place and a voice (p. 46). On the other hand, individuals mention that when discussing inclusion, one cannot make distinctions or segregate based on characteristics, given the intrinsic differences among all people and the need to value each. Additionally, they question the concept of normality as a category that establishes a norm about what is acceptable or expected. Both opinions align with Tiernan’s [15] argument regarding those who advocate for “total inclusion,” placing diversity at the center of the education system. However, these tensions regarding who should be the subjects of inclusion and certain concepts like normality also imply a tension in the concept of inclusion. For this concept to exist, exclusion must also be present, as highlighted by some authors who define them as interrelated and dependent processes of “inclusion/exclusion” [48,49]. Thus, the concept of inclusion would set a standard of “normality” that excluded groups that are expected to adapt to be considered included [49–51]. From this perspective, a hegemonic group would define who should be included or subjects of inclusion and who should not be. In this context, the following question arises: Is the concept of inclusion the most relevant for promoting participatory and equitable environments with a sense of belonging and valuing the inherent diversity of all individuals? This question becomes especially relevant when it is evident that, in the participants’ discourse, the concept is sometimes ambiguous, and they even propose alternatives based on good treatment, coexistence, and pluralism. From this perspective, a hegemonic group would define who should be included or subjects of inclusion and who should not be. In this context, the following question arises: Is the concept of inclusion the most relevant for promoting participatory and equitable environments with a sense of belonging and valuing the inherent diversity of all individuals? This question becomes especially relevant when it is evident that the concept is sometimes ambiguous in the participants’ discourse, and they even propose alternatives based on good treatment, coexistence, and pluralism. In addition to the above, some opinions raise doubts about who should be the subjects of inclusion. This perception arises from recognizing the large number of groups that need inclusion and the sense of a challenge that the institution considers too ambitious and unrealistic. This aligns with the findings of Kauffman, Ward, and Badar [52], who suggest the sense of “illusion” that some people have about total inclusion, as they do not trust that it provides the support students need [16].

All these opinions, including those related to the lack of clarity, knowledge, and ambiguous feelings about the concept of inclusion, align with findings that indicate a need for more consensus in detailing what inclusive education is and what it means to act inclusively [2]. Similarly, the controversy around conflicting opinions on what constitutes educational inclusion [15], coupled with the absence of a strict conceptual focus on this concept [2], corresponds with the expressed need among study participants to define at the institutional level what inclusion means and determine the perspective from which to address it. This is essential to advance in its implementation. Following this line, Slee [53] discusses the lack of understanding and misuse of the term that affects its implementation. He emphasizes the importance of a pragmatic understanding of total inclusion to develop inclusive education [15] successfully.

Beyond the evident lack of clarity surrounding the concept of inclusion and its consequences, observers can also note that shared opinions focus on the individualities and types of diversities within the educational community. As mentioned earlier, this aligns with the logic of policies of differences or identity [47]. Only some of the accounts point to a comprehensive approach to the inclusive culture of the entire institution. Regarding the previous, it is essential to note that while those who participated in the study mentioned the need for an institutional definition to create an inclusive educational community, it is not possible to find consensus based on a single approach, such as the creation of an inclusive community from the proposal of Göransson and Nilholm [19]. This is because the central aspect of this category would focus on the characteristics of the culture/group as a whole rather than solely on the situation of individual subjects. In this regard, it is worth noting that Rengifo [54] raises a primary concern about diversity recognition policies. Collectives may perceive their experiences as unique, and those outside the collective may struggle to understand their ultimate identity. This difficulty inhibits the establishment of common grounds for collective action. In this direction, Knight and Pearl [55] consider that the postmodern “celebration of differences” and increasingly smaller subdivisions result in segregation among groups, impeding communication. According to these authors, the emphasis on individual differences and identities opposes the intention to build a collective based on shared values [49]. Considering the above and recognizing the risks linked to focusing on subdividing each group, we face the challenge of creating institutional spaces for encounters, dialogue, and the development of inclusion definitions. Each educational community should actively embrace these definitions according to its context and uniqueness. This approach actively promotes finding common ground over the differences in individual identities.

## Limitations

Regarding the research’s limitations, we conducted it with a sample from only one higher education institution. Therefore, we need to expand the study to include other academic institutions. Furthermore, as this study is part of a larger project, we recommend that future research focus on converging the conceptualization of educational inclusion.

Although, this limitation allowed for a deep and contextualized analysis of the tensions surrounding the conceptualization of inclusion in a specific environment, limits the generalization of the findings. Each Higher Education Institution (IES) has unique characteristics regarding policies, organizational culture, and community composition, factors that influence the way educational inclusion is understood and applied. Therefore, future research should expand the sample to a more diverse set of institutions representing different regions, cultures, and socioeconomic contexts, which would enrich and complement the findings of this study. Other possible limitations relate to the focus groups, where participants might be influenced by their peers or intimidated by the presence of researchers. To reduce this possibility, the groups were led by expert professionals with experience in this methodology. The temporality of the research could also be a limitation, as the results reflect a specific context and may not be applicable in the near future due to social, political, and educational changes that may occur and that, indeed, have been observed throughout history. Therefore, it is suggested to conduct follow-up studies that can account for the evolution of educational inclusion in university contexts over time.

On the other hand, since this study is part of a larger project, it is suggested that future research continues to focus on the convergence of the conceptualization of educational inclusion, complementing the findings with quantitative and mixed approaches.

To mitigate the limitations inherent in qualitative research, we have maintained commitment and rigor throughout all stages of the study, demonstrated by the formation of a team of expert researchers, both in the research topic and in the methodologies used. This allowed for an in-depth collection, analysis, and interpretation of data with a reflective approach, contributing new knowledge that invites further exploration and advancement in the themes analyzed. With this, the value of this research is demonstrated, aligning with the criteria set by Yardley (2017) [56] for conducting proper and quality qualitative research.

## Conclusions

This study aimed to describe the relationship between the diversity of actors in the university community and their perception of institutional inclusion.

Despite the difficulty expressed by participants in defining inclusion in higher education institutions, it is possible to agree that there is a need to create participatory definitions of the concept of inclusion at the local level. This should be done based on the relevance and stage of advancement of each institution’s inclusive processes.

On the other hand, the proposal emerging from the results analysis of this study suggests that inclusion in higher education should, on the one hand, have a broad approach promoting an inclusive organizational culture shared by all members of the educational community. In this culture, everyone should feel a part, valuing all forms of diversity. Additionally, institutions should strongly complement this approach with specific support or actions to eliminate barriers to the education and participation of individuals from specific historically disadvantaged groups. It is necessary to ensure equity and equal opportunities.

Finally, given the evident tension in the conceptualization, some questions arise that could guide future research: Is the term “inclusion” the most appropriate? What other terms are related to this concept? How does it behave in practice? Is it possible to move away from the logic of exclusion and abnormality while using this term? What are the consequences, and what would be the most suitable term? Overall, this study aims to contribute to developing the debate and critical theorization of the concept of inclusive education in higher education.
